# Glutamate promotes nucleotide synthesis in the gut and improves availability of soybean meal feed in rainbow trout

**DOI:** 10.1186/s40064-016-2634-2

**Published:** 2016-07-08

**Authors:** Chika Yoshida, Mayumi Maekawa, Makoto Bannai, Takeshi Yamamoto

**Affiliations:** Frontier Research Labs, Institute for Innovation, Ajinomoto Co., Inc., 1-1 Suzuki-cho, Kawasaki-ku, Kanagawa 210-8681 Japan; Material Development and Application Labs, Research Institute for Bioscience Products and Fine Chemicals, Ajinomoto Co., Inc., 1-1 Suzuki-cho, Kawasaki-ku, Kanagawa 210-8681 Japan; Feed Group, Tamaki Laboratory, National Research Institute of Aquaculture, Fisheries Research Agency, Tamaki, Mie 519-0423 Japan; Strategy Implementation Group, Business Strategy and Planning Department, Ajinomoto Animal Nutrition Group, Inc., Tokyo, 104-0031 Japan

**Keywords:** Fishmeal alternatives, Feed ingredient, Amino acids

## Abstract

Glutamate (Glu) plays various roles directly or through conversions to other amino acids in intracellular metabolisms such as energy source for enterocytes and precursor for nucleic acids. In this study, we examined the effect of single and chronic oral administration of Glu on cell proliferation in intestine and growth in rainbow trout fed soybean meal (SBM) based diet. In the single dose study, 30, 120 and 360 min after oral administration of 50 and 500 mg/kg Glu, the blood and intestine tissues were collected for amino acid concentration and gene expression analysis. Cell-proliferation was detected 24 h after administration using bromo-deoxy uridine (BrdU) in intestine. In the chronic experiment, fish were fed SBM-based diet added 1 and 2 % of Glu for 8 weeks. Final body weight, plasma amino acid concentrations, gene expression and cell-proliferation in the intestine were analyzed. The expressions of some nucleic acid-synthesis related genes were significantly increased 30 min after administration of 50 mg/kg of Glu. After 8 weeks of feeding, the fish fed SBM-based diet showed significantly lower body weight and microvillus thickness in proximal intestine. Supplementation of 2 % of Glu in the SBM-based feed improved both of them. Though it was not significant difference, Glu tended to increase cell-proliferation in the proximal intestine dose-dependently in both single and chronic administration. Our experiment indicates that Glu has positive effect on rainbow trout fed SBM-based feed by reforming proximal intestine through altering cell-proliferation.

## Background

Glutamate (Glu) is a nonessential amino acid which universally exists in living organism. It plays various roles directly or through conversions to other molecules in cell metabolisms and physiology. Glu serves as an energy source for enterocytes (Burrin and Stoll 2009; Blachier et al. 2009), an excitatory neurotransmitter in the enteric nervous system (Kirchgessner 2001; Cartmell and Schoepp 2000; Zhang et al. 2013) and as a precursor for other biologically active molecules such as glutathione (Newsholme et al. 2003; Reeds et al. 2000). Supplementation of Glu into an enteral diet increases glutathione levels in jejunal mucosa of burned rat (Hasebe et al. 1999). The carbon skeleton of Glu are metabolized mainly into CO_2_, lactate, or alanine and the nitrogen in Glu was utilized for the synthesis of other amino acids such as glutamine (Gln), proline, and arginine (Blachier et al. 2009; Newsholme et al. 2003; Reeds et al. 2000; Nakamura et al. 2013). Gln, in turn, has various functions in cellular metabolism such as a precursor for purine and pyrimidine nucleotide, NAD^+^, and aminosugar or as an energy fuel (Blachier et al. 2009; Newsholme et al. 2003). Although small intestine uses both Glu and Gln as energy fuel, the supply source of each molecule is different. While both dietary and arterial Gln are recruited into intestinal cells. On the other hand, almost all Glu utilized in the gut comes from the lumen (Windmueller and Spaeth 1980; Wu 1998). Glu, as a part of Glutathione synthesized at intestinal mucosa also derived largely from enteral Glu rather than arterial Glu (Reeds et al. 2000). In piglet guts, it is shown that 95 % of the dietary Glu presented to the intestinal mucosa is metabolized in the intestinal cell, and that Glu is the largest resource of energy (Reeds et al. 2000). Although Glu is nonessential amino acid which can be synthesized in the animal body, these studies indicate that utilization of dietary Glu in the gut has an important and particular role in the context of gut and systemic metabolism.

Motivated by the studies on these various functions in cell metabolism, several approaches have been made to utilize Glu as a solution for physiological problems. The most investigated application is for post-weaning piglet, since the intestinal tract of weaning period is not fully developed, and its digestive and absorption capability is poor (Berseth 1996). Besides, it is vulnerable to mechanical damage and infectious disease (Lallès et al. 2007). Thus, weaning piglet often goes into growth failure and is susceptible to diarrhea (Pluske et al. 1997). Supplementation of Glu has been shown to improve morphological abnormality in the small intestine, increase weight gain and feed efficiency, and reduce the occurrence of diarrhea (Rezaei et al. 2013; Wu et al. 2012). Glu increases gene expression of proliferating cell nuclear antigen (PCNA) in jejunum, indicating that it up-regulates cell proliferation (Wu et al. 2012). It is also shown that mixture of Glu and Gln has positive effect on weight gain and increases intestinal villus height in fish: tilapia (Da Silva et al. 2010). However, the primary effect of Glu on the gut health is poorly investigated so far.

In the aquaculture field, reduction of fishmeal in the feed has become inevitable issue because of the growth of aquaculture production and the rising market of fishmeal. Soybean meal (SBM) is one of the promising protein source for fish feed in the future. However, SBM is reported to have some anti-nutritional factors and suppress fish growth (Chikwati et al. 2012; Iwashita et al. 2008; Yamamoto et al. 2010). Among the negative impacts of SBM, tissue degeneration in the distal intestine is one of the most prominent abnormalities in salmonid fish (Suzuki and Yamamoto 2004; Buttle et al. 2001; Krogdahl et al. 2003; Burrells et al. 1999). Since cell turnover should be enhanced in degenerated tissue (Sanden et al. 2005), the energy consumption and biosynthesis of nucleic acids are estimated to be up-regulated. Thus, supplementation of Glu, which is main energy source in the intestine and provides nucleotide precursor thorough the conversion to Gln, to the SBM feed is expected to help restoration from the intestinal damage and contribute to growth promotion.

The aim of this study is to demonstrate the acute effect of oral Glu administration on cell proliferation in the gut (experiment 1) and then examine the chronic effect of Glu supplementation in the SBM feed on growth performance (experiment 2). In the experiment 1, we investigated the effect of single oral Glu administration on nucleotide-biosynthesis related gene expression and quantified cell proliferation by histological analysis. In the experiment 2, we investigated the growth performance, in addition to alteration of basal gene expression and cell proliferation in the gut after 8 weeks of Glu-supplemented feed.

## Methods

### Fish husbandry

Juvenile rainbow trout *Oncorhynchus mykiss* were purchased at Shiga Prefectual Samegai Trout Farm (Maibara, Shiga, Japan). The fish were fed a commercial trout feed (Nippon Formula Feed, Kanagawa, Japan) supplemented with 5 % Pollock oil twice a day to apparent satiation for 1 month. Then, fish were anesthetized in 100 mg/L ethyl 3-aminobenzoate methanosulfonate (Tricaine, Sigma Aldrich, St. Louis, USA) and weighed individually after 48 h-fasting. The fish were sorted into a uniform size (25.3, 9.53 g mean body weight (BW) in experiment 1, 2 respectively) and kept for 10 days for naturalization.

In experiment 1, the fish were anesthetized by Tricaine and 50 or 500 mg kg/BW mL Glu (suspended in 4 % carboxymethyl cellulose) were administered per os. Five fish from each group were additionally administered 50 mg kg/BW of bromo-deoxy-uridine (BrdU, Becton, Dickinson and Company, California, USA) intraperitoneally. Before or 30, 120, and 360 min after Glu administration, fish without BrdU injection were anesthetized again by Tricaine (6 fish for each time) and blood was collected using an ethylenediaminetetraacetic acid (EDTA) coated syringe for determination of plasma amino acids concentrations. Then, fish were sacrificed. The proximal and distal intestines were dissected out and immersed into RNA later (Thermo Fisher Scientific, Massachusetts, USA) to preserve RNA. The fish administered BrdU were sacrificed 24 h after the administration. Proximal and distal intestines were dissected and were immersed in 10 % phosphate buffered formarin (pH 7.4, Wako Pure Chemical Industries, Osaka, Japan) to fix the tissues.

In experiment 2, 52–53 fish per group were kept in 60 L volume tanks with water supply of 3 L/min. The water temperature was 15.6 ± 0.3 °C throughout the experiment. The fish were acclimatized to the experimental conditions and SBM-based diet for 10 days before the experiment. Then the experimental diet was fed by hand to apparent satiation twice a day, 6 days a week for 8 weeks. The fish were fasted for 48 h before the termination of the experiment and 8 fish from each tank were administered 50 mg kg/BW of BrdU intraperitoneally under anesthesia 24 h before the termination of the experiment. At the time of terminal BW measurement, 8 fish (without BrdU injection) from each tank were taken and blood was collected. Then the fish were sacrificed and the liver and gallbladder were dissected out and weighed. Subsequently, proximal and distal intestines were collected. The fish which were injected BrdU were sacrificed at the same time and intestines were collected.

### Experimental diet

In experiment 2, four iso-nitrogenous diets were prepared (Table 1). Chilean mackerel meal was used as the main protein source in the positive control diet (diet FM). In the three non-fish meal diets, SBM at a level of 49.7 %, together with corn gluten meal at a level of 17.11 % were included as the main protein sources (SBM, Glu 1 %, Glu 2 %). Diet Glu 1 %, Glu 2 % were supplemented crystalline Glu 1 %, 2 % wet weight respectively. Gelatin was used to balance the proportion of the ingredients among non-fish meal diets. To the non-fish meal diets, methionine and lysine were supplemented to simulate the digestible contents of diet FM (Yamamoto et al. 2002). Level of fish oil and soybean meal oil were adjusted to provide a diet with lipids from fish and those from vegetables being at ratio of 2:1. The ingredients were mixed well and made into dry pellets using a laboratory pellet mill (California Pellet Mill, San Francisco, CA, USA). The pellets were dried for 4 h at 60 °C and stored at −20 °C until use.

**Table 1:** Composition and nutritional contents of the experimental diet for rainbow trout in experiment 2

Ingredients (g/kg feed)	FM	SBM	Glu 1 %	Glu 2 %
Fish meal	480	0	0	0
Soybean meal	40	497	497	497
Corn gluten meal	20	171.1	171.1	171.1
Wheat flour	180	60	60	60
Gelatin	0	20	10	0
Fish oil	55.4	100.1	100.2	100.4
Soybean oil	28.3	20.4	20.8	21.1
α-Starch	60	28	28	28
Methionine	0	4.2	4.3	4.4
Lysine-HCl	0	12.2	12.7	13.3
Glutamate	0	0	10	20
Betaine	0	5	5	5
Vitamin mix	5	5	5	5
Choline chloride	2.5	2.5	2.5	2.5
Mineral mix	30	50	50	50
Cellulose	98.8	24.5	23.4	22.2
Total	1000	1000	1000	1000
Analytical contents (wet basis, %)				
Aspartic acid + asparagine	3.61	3.45	3.39	3.35
Threonine	1.67	1.37	1.32	1.32
Serine	1.68	1.93	1.85	1.83
Glutamic acid + glutamine	5.90	7.17	8.10	8.94
Glycine	2.34	1.84	1.59	1.34
Alanine	2.46	2.19	2.08	2.04
Valine	1.91	1.61	1.62	1.61
Isoleucine	1.63	1.50	1.53	1.51
Leucine	3.07	3.72	3.67	3.68
Tyrosine	1.31	1.49	1.47	1.49
Phenylalanine	1.66	1.95	1.95	1.92
Histidine	1.53	0.90	0.87	0.86
Lysine	2.86	2.65	2.68	2.67
Arginine	2.39	2.25	2.16	2.10
Proline	1.87	2.55	2.38	2.23
Cystine	0.40	0.54	0.53	0.55
Methionine	1.04	1.03	1.04	1.05
Tryptophan	0.477	0.378	0.385	0.381
Crude protein (%)	41.7	39.0	39.0	38.3
Moisture (%)	6.5	8.8	7.8	8.6

### Plasma amino acid analysis and histological analysis

For plasma amino acid analysis, the collected blood was centrifuged to obtain plasma (4 °C, 20 min, 2000*g*) and kept at −80 °C until analysis. To prepare sample for analysis, the plasma was mixed with 2 volumes of 5 % (w/w) trichloroacetic acid, and centrifuged immediately (4 °C, 20 min, 8000*g*) to remove precipitated protein. All samples were kept at 4 °C during all steps to minimize chemical reactions of thiol metabolites. The amino acid concentrations were measured by an automatic amino acid analyzer (L-8800; Hitachi, Tokyo, Japan). Briefly, amino acids, separated by cation exchange chromatography, were detected spectrophotometrically after post column reaction with ninhydrin reagent.

For histological analysis, intestine samples were fixed in 10 % phosphate-buffered formalin (pH 7.4). The fixed tissues were dehydrated in ethanol, equilibrated in xylene and embedded in paraffin according to standard histological techniques. For morphological observation, 4 μm of sections were cut out and stained with hematoxylin and eosine. Microvillus thickness was measured using calculating software (Sensiv Measure; Mitani Corporation, Tokyo, Japan) under microscope as the two-point distance of villus root. The microvillus thickness for each fish was calculated as the average of 5 microvilli per fish. For immunohistochemical detection of BrdU, 4 μm sections were cut out and stained using BrdU in situ detection kit (Becton, Dickinson and Company) following the manufacturer’s instructions. The number of BrdU-positive cells per microvillus for each fish was counted and calculated as the average of BrdU positive cells in 5 microvilli per fish.

### Quantitative real-time PCR

Total RNA was extracted from the homogenized intestine using an RNeasy kit (Qiagen, California, USA) following the manufacturer’s instructions. Equal amounts (2 ng) of RNA were reverse-transcribed using Omniscript RT kit (Qiagen) as per the manufacturer’s instructions. Primers for quantitative RT-PCR were designed in reference to the sequence in Genbank (https://www.ncbi.nlm.nih.gov/genbank/) using the primer design software Primer3 (http://frodo.wi.mit.edu/). As for the genes with no reference on Genbank, expressed sequence tags (EST) similar to the known base sequence of teleost were searched from EST database by BLAST search (offered by The Computational Biology and Functional Genomics Laboratory of Dana-Farber Cancer institute and Harvard School of public Health (http://compbio.dfci.harvard.edu/tgi/cgi-bin/tgi/Blast/index.cgi). Quantitative RT-PCR was performed on ABI Prism^®^ 7900 Sequence Detection System (Life Technologies, California, USA), with SYBR select PCR master mix (Life Technologies, California, USA) following the manufacturer’s instructions. The PCR protocol consisted of one 10-min denaturation cycle at 95 °C followed by 40 cycles of denaturation at 95 °C for 15 s and finally annealing/extension at 60 °C for 1 min. Standard curves for each gene were obtained. All quantitative RT-PCR data were expressed as relative mRNA levels after normalizing to 60S acidic ribosomal protein P0 (arbp). The primer pair sequences and the reference base sequence for BLAST search are listed in Tables 2 and 3, respectively.

**Table 2:** Primer sequences used for qPCR analysis

Gene symbol	Gene name	Sequence 5′–3′	Product size (bp)	Gene ID^a^
arbp	60S acidic ribosomal protein P0	F: GCTGTAAAAGCGATCCTTCG	135	TC209405
		R: ATTGTCTGCACCCACAATGA		
cps2	Carbamoyl-phosphate synthetase II	F: ACATCCAGTACGCCCTCAAC	181	NCBI Ref. Seq.: AF014386.1
		R: GTACTTGGCACCGATGGACT		
		R: AAGTCCGCCTGTCTGAGTGT		
pfas	Phosphoribosylformylglycinamidine synthase	F: TGACGATACGCTGAGTCTGG	163	TC176935
		R: GGCCTTGATCCCTACACTGA		
ppat	Phosphoribosyl pyrophosphate amidotransferase	F: ACATCCTACTCGCTGCTGGT	195	TC216500
		R: GTACTTGGCACCGATGGACT		

^a^Gene IDs with TC are EST of DFCI R.trout Gene Index

**Table 3:** Reference sequences used for EST search

Gene ID (gene symbol)	Reference sequence (upper: protein, lower: mRNA)	Identity
TC176935 (pfas)	Phosphoribosylformylglycinamidine synthase [*Danio rerio*] (NP_001038667.1)	267/360 (74 %)
	Danio rerio phosphoribosylformylglycinamidine synthase (pfas), mRNA (NM_001045202.1)	746/1036 (72 %)
TC216500 (ppat)	Amidophosphoribosyltransferase [*Salmo salar*](NP_001133427.1)	424/438 (97 %)
	Salmo salar Amidophosphoribosyltransferase (pur1), mRNA	1446/1532 (94 %)

### Statistics

The data are presented as the mean ± SEM. Data were analyzed by one-way ANOVA to determine main effects of treatment. In case normality test failed, one-way ANOVA on ranks was used. In experiment 1, Dunnett’s post hoc test was used to identify statistical differences between initial group and treatment groups. In experiment 2, Tukey post hoc test was used for all multiple comparison procedure. Probability level of <0.05 was considered as significant.

## Results

### Experiment 1

#### Plasma amino acid concentration

The free amino acid concentrations in plasma are shown in Table 4. Plasma Glu concentrations significantly increased at 30, 120, and 360 min after 500 mg/kg Glu administration and they returned to basal level 24 h after administration. Plasma Glu concentrations in the group administered 50 mg/kg were not significantly different from the basal level. Plasma concentrations of neither Gln, alanine (Ala) nor aspartate (Asp), the amino acids which are directly generated from Glu in intestine, were significantly changed to the basal level throughout the experimental period in both 50 and 500 mg/kg administered fish (Table 4).

**Table 4:** Plasma amino acid concentrations by oral Glu administration (nmol/mL)

	0 h	30 min		120 min	
		50 mg/kg	500 mg/kg	50 mg/kg	500 mg/kg
Thr	170.4 ± 47.3	176.2 ± 37.9	177.0 ± 25.9	163.2 ± 32.6	223.2 ± 57.7
Ser	85.6 ± 23.9	117.9 ± 41.9	147.2 ± 43.3	96.8 ± 28.9	123.8 ± 18.8
Gly	290.5 ± 109.0	321.0 ± 161.9	410.6 ± 59.2	294.3 ± 104.4	390.3 ± 104.5
Ala	331.8 ± 82.0	428.2 ± 112.9	531.8 ± 82.8	358.6 ± 76.7	534.6 ± 97.4
Gln	221.9 ± 73.9	241.6 ± 80.1	288.7 ± 31.1	193.1 ± 55.7	282.2 ± 60.4
Glu	45.6 ± 5.1	92.3 ± 43.9	115.0 ± 38.0*	62.7 ± 22.9	406.6 ± 354.7*
Asn	73.8 ± 12.5	75.0 ± 21.6	93.9 ± 14.6	75.9 ± 17.1	77.3 ± 23.4
Asp	17.9 ± 6.1	17.1 ± 9.1	16.9 ± 8.0	14.2 ± 5.3	29.8 ± 10.9
Arg	88.3 ± 10.3	94.5 ± 30.9	92.6 ± 7.7	87.1 ± 3.9	95.4 ± 27.4
Lys	333.0 ± 84.5	452.5 ± 385.5	244.0 ± 28.7	282.8 ± 88.7	265.9 ± 101.2
Val	388.3 ± 131.4	512.3 ± 276.6	287.9 ± 21.3	372.1 ± 188.1	322.0 ± 55.7
Ile	165.6 ± 76.1	236.8 ± 145.7	120.3 ± 9.4	162.6 ± 106.6	141.4 ± 32.5
Leu	275.8 ± 109.4	371.2 ± 203.8	203.7 ± 17.4	266.7 ± 151.0	239.6 ± 54.2
Tyr	49.1 ± 8.2	69.6 ± 10.4*	72.9 ± 7.8*	64.4 ± 9.2	69.9 ± 10.3*
Phe	90.7 ± 15.1	115.7 ± 11.9*	111.8 ± 11.5	105.6 ± 10.4	104.9 ± 17.8
Cys	11.4 ± 2.3	13.0 ± 3.6	15.5 ± 2.0	12.3 ± 1.9	13.9 ± 1.9
Met	72.4 ± 8.5	82.2 ± 20.5	96.4 ± 9.5	80.6 ± 14.0	86.1 ± 6.7
Trp	49.0 ± 6.6	62.0 ± 3.7	64.2 ± 4.1*	54.1 ± 13.4	64.8 ± 7.6*
His	98.9 ± 21.7	121.4 ± 37.2	169.3 ± 19.3*	119.0 ± 34.0	163.4 ± 36.3
Pro	32.3 ± 7.8	43.6 ± 4.9	41.7 ± 3.4	34.2 ± 5.1	45.7 ± 9.4

	360 min		1440 min		
	50 mg/kg	500 mg/kg	50 mg/kg	500 mg/kg	
Thr	184.6 ± 22.7	184.4 ± 38.5	291.7 ± 119.2	214.8 ± 74.0	
Ser	113.6 ± 26.9	104.4 ± 48.1	146.8 ± 65.3	108.0 ± 46.7	
Gly	360.9 ± 103.0	369.6 ± 143.5	503.5 ± 323.1	377.4 ± 303.5	
Ala	407.1 ± 74.8	667.9 ± 250.8	328.2 ± 84.5	386.5 ± 216.3	
Gln	215.4 ± 41.2	259.3 ± 95.6	302.4 ± 135.1	262.1 ± 150.0	
Glu	89.3 ± 40.8	2309.0 ± 1729.4*	66.2 ± 16.5	61.3 ± 22.4	
Asn	77.0 ± 14.8	77.4 ± 17.9	107.9 ± 33.2	85.2 ± 35.8	
Asp	20.8 ± 7.9	86.0 ± 60.7	19.7 ± 5.9	14.2 ± 4.3	
Arg	125.8 ± 23.6	89.7 ± 20.4	156.9 ± 55.4	91.7 ± 22.5	
Lys	385.9 ± 145.9	287.9 ± 151.8	518.5 ± 152.3	308.7 ± 77.3	
Val	405.4 ± 124.2	350.7 ± 108.2	499.9 ± 117.6	513.1 ± 144.9	
Ile	183.5 ± 58.1	156.8 ± 45.7	217.1 ± 55.9	207.3 ± 65.1	
Leu	308.6 ± 95.3	263.0 ± 75.7	348.2 ± 73.2	341.9 ± 103.5	
Tyr	87.2 ± 15.3*	71.6 ± 7.4*	72.1 ± 13.3*	57.7 ± 6.1	
Phe	120.3 ± 19.0*	104.6 ± 5.7	124.9 ± 15.5*	105.6 ± 15.1	
Cys	14.0 ± 2.8	17.7 ± 2.9*	18.9 ± 3.5	18.3 ± 4.9	
Met	88.7 ± 6.4	83.3 ± 11.1	98.0 ± 21.4	86.2 ± 16.1	
Trp	65.1 ± 6.9*	60.0 ± 12.8	64.3 ± 10.7*	59.4 ± 5.7	
His	130.1 ± 29.0	121.0 ± 24.4	252.5 ± 166.7*	126.4 ± 61.3	
Pro	69.6 ± 16.0*	50.8 ± 15.0	61.7 ± 27.8*	43.1 ± 9.0	

Variation in plasma amino acid concentrations after oral Glu administration. The blood were collected before (0 min; initial), 30, 120, 360 and 1440 (24 h) min after administration of 50, 500 mg/kg Glu

* p < 0.05 compared to initial group

#### Gene expression

**Fig. 1:**
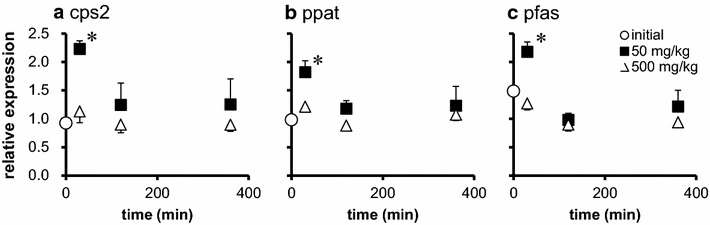
Alterations of gene expressions in proximal intestine by oral Glu intake. Relative expression of genes related to nucleic acid synthesis in proximal intestine. **a** cps2: carbamoyl-phosphate synthetase, **b** ppat: phosphoribosyl pyrophosphate amidotransferase, **c** pfas: phosphoribosylformylglycinamidine synthase. The tissues were collected before (initial), 30, 120, and 360 min after administration of 50 or 500 mg/kg Glu. The expression of the genes were estimated by quantitative RT-PCR and normalized by arbp. All values are expressed as the mean ± SEM (n = 6). *p < 0.05 as compared to initial group

**Fig. 2:**
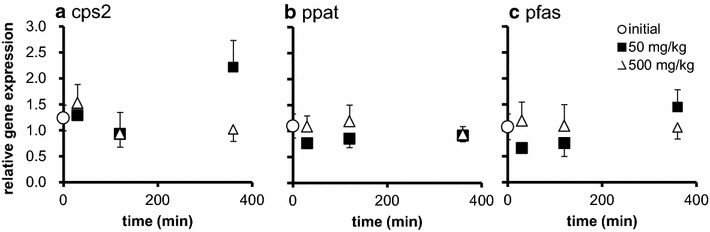
Alterations of gene expressions in distal intestine by oral Glu intake. Relative expression of genes related to nucleic acid synthesis in distal intestine. **a** cps2: carbamoyl-phosphate synthetase, **b** ppat: phosphoribosyl pyrophosphate amidotransferase, **c** pfas: phosphoribosylformylglycinamidine synthase. The tissues were collected before (initial), 30, 120, and 360 min after administration of 50, 500 mg/kg Glu p.o. The expression of the genes were estimated by quantitative RT-PCR and normalized by arbp. All values are expressed as the mean ± SEM (n = 6)

The expressions of genes related to nucleic acid synthesis in proximal and distal intestine are shown in Figs. 1 and 2 respectively. The expression of carbamoyl-phosphate synthetase2 (cps2), phosphoribosyl pyrophosphate amidotransferase (ppat), phosphoribosylformylglycinamidine synthase (pfas) were significantly increased 30 min after 50 mg/kg Glu administration compared to the initial group, returning to basal level after 120 min (Fig. 1). The expressions of those genes were not significantly different in the proximal intestine of 500 mg/kg Glu group and distal intestine of both 50 and 500 mg/kg Glu group compared to the initial group (Figs. 1, 2).

#### Histological analysis

**Fig. 3:**
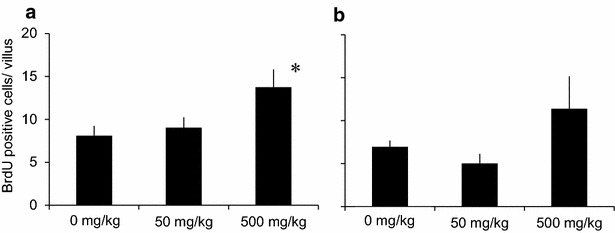
Effects of Glu intake on cell proliferation in the intestine. The number of BrdU positive cells per microvillus in **a** proximal intestine, **b** distal intestine. The tissues were collected 24 h after 0, 50, 500 mg/kg Glu (p.o.) and BrdU (i.p.) administration. Data are shown as the average of five villi from each fish. *p < 0.05 as compared to 0 mg/kg group

Figure 3 shows the number of BrdU positive cells in proximal and distal intestine. In proximal intestine of fish administered 500 mg/kg Glu, the number of BrdU positive cells significantly increased compared to 0 mg/kg group. In distal intestine there was no significant difference between each groups.

### Experiment 2

#### Growth and feed performance

**Fig. 4:**
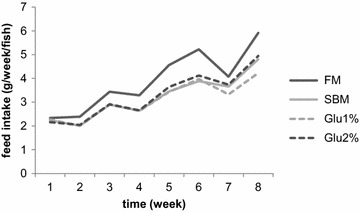
Weekly feed intake. The experimental diets were fed by hand to apparent satiation twice a day, 6 days a week for 8 weeks. Data are shown as week total intake per fish

Weekly feed intake is shown in Fig. 4 and results of the growth and feed performance are shown in Table 5. The fish ate more FM-based feed than SBM-based feeds throughout the experimental period. Among three SBM-based feeds, there was no big difference in feed intake (Fig. 4). The fish fed SBM and Glu 1 % showed significantly lower final body weight compared to the fish fed FM. There was no significant difference between FM and Glu 2 % groups in the final body weight. Hepato-somatic index (HIS) and gallbladder-somatic index (GBSI) of the fish fed SBM, Glu 1 % and Glu 2 % were significantly lower compared to the fish fed FM.

**Table 5:** Fish growth and performance of four experimental feeds

	FM	SBM	Glu 1 %	Glu 2 %
Initial BW (g)	9.56 ± 0.51	9.73 ± 0.56	9.60 ± 0.49	9.70 ± 0.52
Final BW (g)	57.80 ± 5.43	41.44 ± 5.13*	40.24 ± 4.41*	47.22 ± 3.84
SGR (%/day)	2.57	2.07	2.05	2.26
Weight gain (%)	504.37	325.98	318.93	387.03
Feed efficiency ratio	1.03	0.85	0.85	0.87
DFC (%BW/day)	2.40	2.53	2.48	2.54
Mortality (%)	0.00	0.00	2.00	0.00
HSI (%)	1.29 ± 0.13	0.98 ± 0.18*	0.97 ± 0.09*	1.05 ± 0.17*
GBSI (%)	0.30 ± 0.06	0.17 ± 0.03*	0.17 ± 0.03*	0.21 ± 0.06*

The fish were challenged to 8 weeks of experiment. The final body weight and tissue weight were measured after fasting for the last 48 h of the experiment. The initial body weight was measured without fasting

*BW* body weight, *SGR* specific growth rate, *DFC* daily feed consumption, *HSI* hepato-somatic index (liver weight (g)/body weight (g)) × 100, *GBSI* gallbladder-somatic index (gallbladder weight (g)/body weight (g))

* p < 0.05 compared to FM group

#### Plasma amino acid concentration

The free amino acid concentrations in plasma are shown in Table 6. Plasma Glu concentrations were not significantly different between groups. Plasma concentrations of Gln were significantly lower in Glu 1 % and Glu 2 % group compared to FM and SBM group.

**Table 6:** Variation in plasma Glu and Gln concentrations by 8 weeks of Glu supplementation in feed

	FM	SBM	Glu 1 %	Glu 2 %
Glu	2.92 ± 0.43	3.27 ± 0.35	3.82 ± 0.38	3.72 ± 0.44
Gln	9.33 ± 0.58	10.17 ± 0.86	3.56 ± 0.25	3.57 ± 0.43
Thr	162.2 ± 15.7	181.6 ± 10.4	179.0 ± 8.5	168.6 ± 21.7
Ser	180.0 ± 9.0	187.4 ± 20.5	163.0 ± 7.2	147.8 ± 13.2
Gly	1004.7 ± 69.0^a^	1169.7 ± 83.4^a^	695.1 ± 40.3^b^	925.1 ± 69.1^ab^
Ala	382.5 ± 22.1^a^	599.2 ± 35.9^b^	557.4 ± 55.5^b^	673.0 ± 93.6^b^
Gln	279.8 ± 17.4^a^	305.2 ± 25.9^a^	106.9 ± 7.4^b^	107.2 ± 12.8^b^
Glu	87.6 ± 12.9	98.0 ± 10.6	114.7 ± 11.3	111.5 ± 13.1
Asn	120.1 ± 12.1^ab^	115.2 ± 17.5^a^	157.2 ± 9.3^ab^	178.2 ± 20.3^b^
Asp	22.2 ± 1.7^a^	68.5 ± 17.7^b^	49.9 ± 9.6^ab^	62.4 ± 7.3^b^
Arg	103.0 ± 8.7	120.5 ± 11.8	126.7 ± 10.2	137.4 ± 14.7
Lys	220.3 ± 20.7	308.7 ± 47.4	286.2 ± 23.4	256.4 ± 28.9
Val	199.6 ± 7.4^a^	291.4 ± 13.9^b^	274.4 ± 15.1^b^	264.7 ± 26.6^ab^
Ile	82.4 ± 3.9^a^	116.0 ± 5.2^b^	117.2 ± 6.3^b^	117.9 ± 14.9^b^
Leu	146.5 ± 6.5^a^	236.2 ± 10.7^b^	257.6 ± 17.9^b^	282.6 ± 42.0^b^
Tyr	53.5 ± 2.6^a^	60.9 ± 4.8^ab^	73.3 ± 4.8^b^	94.5 ± 10.5^b^
Phe	108.5 ± 3.7	120.7 ± 5.4	116.7 ± 6.8	146.3 ± 16.2
Cys	13.0 ± 0.6	12.0 ± 0.6	14.0 ± 0.8	15.9 ± 2.2
Met	74.9 ± 2.8	92.7 ± 4.0	87.8 ± 3.7	95.2 ± 10.0
Trp	32.6 ± 0.8^a^	26.4 ± 1.0^ab^	20.5 ± 0.9^b^	21.6 ± 2.4^b^
His	188.8 ± 10.7^a^	160.4 ± 10.7^ab^	138.1 ± 5.1^b^	136.8 ± 9.8^b^
Pro	40.1 ± 2.5^a^	82.4 ± 9.1^b^	86.0 ± 8.7^b^	136.7 ± 29.5^b^

The fish were challenged to 8 weeks of experiment. The blood was collected after fasting during the last 48 h of the experiment. Different letters indicate significant difference between groups (p < 0.05)

#### Gene expression

**Fig. 5:**
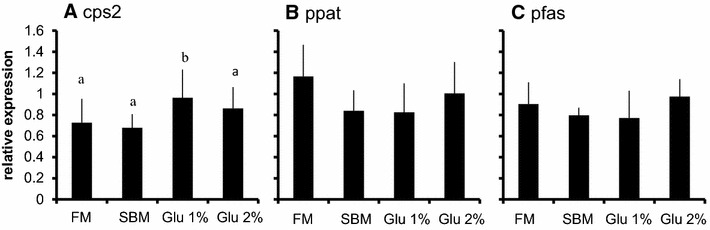
Alterations of gene expressions in proximal intestine by 8 weeks of Glu supplementation. Relative expression of genes related to nucleic acid synthesis in proximal intestine. **A** cps2: carbamoyl-phosphate synthetase, **A** ppat: phosphoribosyl pyrophosphate amidotransferase, **C** pfas: phosphoribosylformylglycinamidine synthase. The fish were challenged to 8 weeks of experiment. The tissues were collected after the 48 h-fasting. The expression of the genes were estimated by quantitative RT-PCR and normalized by arbp. All values are expressed as the mean ± SEM (n = 8). *Different letters* indicate significant difference between groups (p < 0.05)

**Fig. 6:**
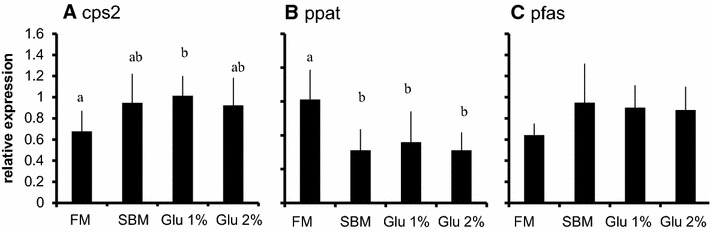
Alterations of gene expressions in distal intestine by 8 weeks of Glu supplementation in feed. Relative expression of genes related to nucleic acid synthesis in distal intestine. **A** cps2: carbamoyl-phosphate synthetase, **B** ppat: phosphoribosyl pyrophosphate amidotransferase, **C** pfas: phosphoribosylformylglycinamidine synthase. The fishes were challenged to 8 weeks of experiment. The tissues were collected after the 48 h-fasting. The expression of the genes were estimated by quantitative RT-PCR and normalized by arbp. All values are expressed as the mean ± SEM (n = 8). *Different letters* indicate significant difference between groups (p < 0.05)

The expressions of genes related to nucleic acid synthesis in proximal and distal intestine are shown in Figs. 5 and 6 respectively. The expression of cps 2 significantly increased in Glu 1 % group compared to SBM group in proximal intestine. Neither expressions of ppat nor pfas showed significant difference between groups (Fig. 5). The expressions of cap 2 in distal intestine also significantly increased in Glu 1 % group compared to SBM group. The expressoin of ppat significantly decreased in the fish fed SBM-based feed (SBM, Glu 1 %, Glu 2 %) compared to the fish fed FM in distal intestine. There was no significant difference in the expression of pfas in distal intestine (Fig. 6).

#### Histological analysis

**Fig. 7:**
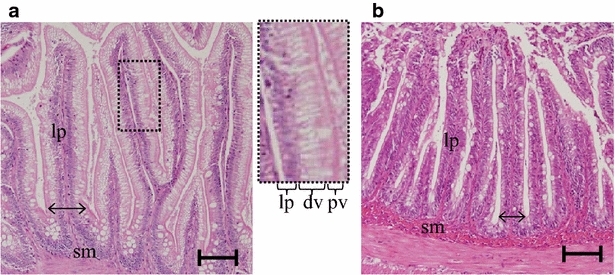
Morphological alteration in distal intestine by SBM feed. Transverse section of distal intestine mucosal fold **a** FM diet group, **b** SBM diet group. The fish were challenged to 8 weeks of experiment and fasted for the last 48 h of experiment. The *central panel* is an extended image of the area surrounded by a broken line in **a**. *dv* digestive vacuole, *lp* lamina propria, *pv* pinocytotic vacuoles, *sm* submucosa. *Scale bar* 100 μm

**Fig. 8:**
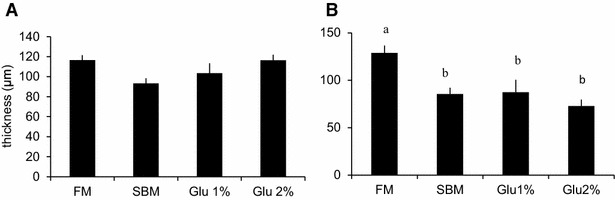
Effects of Glu intake on microvilli thickness in the proximal intestine. The thickness of microvillus of proximal intestine. The fish were fasted for the last 48 h of the experiment. All values are expressed as the mean ± SEM (n = 8). *Different letters* indicate significant difference between groups (p < 0.05)

**Fig. 9:**
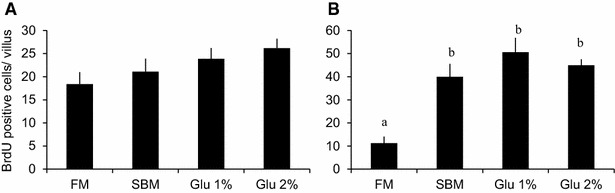
Effects of Glu intake on cell proliferation in the intestine. The number of BrdU positive cells per microvillus of **A** anterior intestine, **B** posterior intestine. The fish were challenged to 8 weeks of experiment. The fish were fasted for the last 48 h of the experiment. BrdU was administered i.p. 24 h before the sacrifice. All values are expressed as the mean ± SEM (n = 8). *Different letters* indicate significant difference between groups (p < 0.05)

Figure 7 shows the optical microscope images of transversal sections of distal intestine. The epithelial cells of mucosal folds in the distal intestine of fish fed SBM showed morphological changes such as absence of granulated pinocytotic vacuoles and thinner villus thickness. Neither the intestine of the fish fed Glu 1 % nor Glu 2 % improved these abnormalities (figure not shown). The villus thickness is shown in Fig. 8. Distal intestine of fish fed SBM feed showed significantly thinner villus. Though it was not significant difference, the villus thickness showed tendency to improve Glu dose-dependently in proximal intestine but not in distal intestine. The number of BrdU-positive cells per villi was counted 24 h after BrdU injection and shown in Fig. 9. There was no significant difference in the number of BrdU positive cells between groups in the proximal intestine though it showed tendency to increase Glu-dose dependently. In distal intestine, the number significantly increased in the SBM-based diet feed groups (SBM, Glu 1 %, Glu 2 %) compared to FM group.

## Discussion

This study demonstrated that single oral administration of glutamate (Glu) altered expressions of genes related to nucleotide synthesis in rainbow trout proximal intestine, and supplementation of 2 % of Glu in the SBM-based feed improved microvillus thickness in proximal intestine and ameliorated body weight gain. Though it was not significant difference, Glu tended to increase cell-proliferation in the proximal intestine dose-dependently. These data indicate that Glu has positive effect on rainbow trout fed SBM-based feed by reforming proximal intestine through altering cell-proliferation.

Previous studies have shown that supplementation of 1–4 % of Glu to the feed up-regulates gene expression of proliferating cell nuclear antigen (PCNA) and increases villus height in jejunum in weaning piglet, resulting in greater growth performance compared to non-supplemented feed (Rezaei et al. 2013; Wu et al. 2012). Glu in combination with glutamine (Gln) is also shown to increase villus height and growth performance in tilapia (Da Silva et al. 2010). In this study, we showed that the positive effect of Glu on intestine could be obtained by its single oral administration. Thirty minutes after 50 mg/kg Glu administration, the expressions of carbamoyl-phosphate synthetase 2 (cps 2), phosphoribosyl pyrophosphate amidotransferase (ppat) and phosphoribosylformylglycinamidine synthasegenes (pfas) were up-regulated (Fig. 1). These genes encode enzymes which catalyze the reactions of importing Gln to purine or pyrimidine base. Although plasma Gln concentrations did not increase significantly (Table 4), Glu can be converted into Gln in one enzymatic reaction (Newsholme et al. 2003) in the intestinal epithelium cells, and converted Gln can enhance up-regulation of these gene expressions. However, such mRNA up-regulations were not seen when fish was administered 500 mg/kg Glu. These results indicate the existence of an upper limit in intracellular Glu (or Gln) concentration which is suitable to up-regulate enzyme reaction. In mammal, majority of orally administered Glu is metabolized in intestinal epithelium cells before reaching portal vein (Reeds et al. 2000; Nakamura et al. 2013; Reeds et al. 1996), however, in our experimental condition, plasma Glu concentration increased sharply 120–360 min after 500 mg/kg Glu administration (Table 4). It is assumed that administered Glu was not metabolized in intestine because of its amount. On the other hand, the number of BrdU positive cells in proximal intestine showed tendency to increase 24 h after 500 mg/kg Glu administration (Fig. 3). Although the suitable intracellular concentration is still unclear, suitable administration concentrations would be exist in the range between 50 and 500 mg/kg.

In contrast, neither mRNA expressions nor BrdU-positive cell number in distal intestine were changed by Glu administration. Glu uptake by enterocyte depends on the existence of suitable transport systems. In mammal, it is reported that transporters responsible for amino acid uptake mainly exist proximal intestine (small intestine; Dave et al. 2004; Broberg et al. 2012). In fish, it is also reported that the absorption rates of amino acids are different from proximal to distal intestine, and proximal intestine uptakes more amino acids than distal intestine in rainbow trout (Bakke-McKellep et al. 2000; Santigosa et al. 2011). It is assumed that insufficient distribution of transporter responsible for Glu import is a reason for ineffectiveness of Glu on distal intestine.

The fish fed SBM diet showed significantly lower final body weight compared to the fish fed FM feed. Since daily feed consumption did not show any significant differences in this study, the direct cause of this growth failure seems to be the decline in feed conversion efficiency. Hepato-somatic index (HSI) and gallbladder-somite index (GBSI) were also significantly lowered in SBM group, suggesting impaired nutritional utilization. Lowered GBSI means decreased bile content because of anti-nutritional factors in SBM (Iwashita et al. 2008; Yamamoto et al. 2007, 2008). Based on the histological observation, the area of lipid droplets in the liver decreased in the fish fed SBM diet (data not shown), similar to previous studies (Suzuki and Yamamoto 2004). The decrease of bile is assumed to cause failure in lipid absorption, leading to less lipid content in the liver. Since lipid is one of major nutrients for fish, failure in its utilization supposedly lowers feed conversion efficiency. The epithelial cells of the distal intestine of fish fed FM diet showed well-developed microvilli and abundant digestive vacuoles. On the other hand, the distal intestine of SBM group showed thinner brush border and less digestive vacuoles, resulting in thinner villi (Fig. 6). Moreover, cell- proliferation in distal intestine was significantly increased in the fish fed SBM diet (Fig. 8), supposedly to recover from damage caused by SBM, as previously reported (Sanden et al. 2005). Neither these abnormalities nor cell-proliferation were altered by supplementation of Glu to SBM. Glu seems little effect on distal intestine even when administered for long term.

In this study, however, supplementation of 2 % of Glu on SBM-based feed improved the restrained weight gain caused by SBM (Table 5). The feed efficiency ratio slightly increased in Glu 2 % group (0.87) compared to SBM group (0.85), and feed intake was also increased in Glu 2 % group (35.8 g per fish) compared to SBM group (34.6 g per fish). Since the typical effects of anti-nutritional factors in SBM, such as lowered HSI/GBSI and abnormality in distal intestine morphologies, showed no improvement by Glu 2 % feed, Glu seems to act on other phenotype. In previous studies, no abnormalities have been reported in proximal intestine in the fish fed SBM-based diet (Suzuki and Yamamoto 2004; Buttle et al. 2001; Krogdahl et al. 2003; Burrells et al. 1999). In this study, however, the thickness of microvilli in proximal intestine not significantly but thinner in SBM group compared to FM group (Fig. 7). It is also reported that both proximal- and distal-intestine nutrient absorption is modified by replacement of fish meal by plant protein (Santigosa et al. 2011). Although there was no other abnormality in morphology like distal intestine, SBM is assumed to have some negative effect on proximal intestine, or the absorption of nutrients in proximal intestine was possibly lowered by SBM. Supplementation of Glu on SBM-diet dose-dependently improved the thickness of microvilli in proximal intestine (Fig. 7). Besides, though it was not significantly different, the number of BrdU positive cells in proximal intestine slightly increased in the fish fed SBM diet compared to FM diet and showed Glu dose-dependent tendency to increase (Fig. 8). It is indicated that Glu helps proximal intestine reform from some mal-effect of SBM. It is simply possible that Glu was absorbed as nutrients and expand the area of digestive vacuole, resulting in thicker microvilli. The absorbed Glu would be utilized as energy or converted into other molecules such as other amino acids, providing necessary elements to intestinal cells. The plasma Gln concentrations in Glu 1 % and 2 % group were significantly lower compared to SBM and FM group. Since plasma Gln is the second largest source of energy for intestine (Reeds et al. 1996, 2000), it indicates that intracellular metabolism in the intestine was activated by Glu supplementation to feed.

In conclusion, we showed that supplementation of 2 % of Glu to SBM-diet for 8 weeks improves growth of rainbow trout through reforming microvilli in proximal intestine. We also demonstrated that single oral Glu administration alters expressions of genes related to nucleotide synthesis in proximal intestine, and at least part of the long-term effect of Glu is indicated to result from up-regulation of cell-proliferation in the proximal intestine. Although further investigation is needed to clear the effects of Glu in fish, Glu has shown to have positive effect on the growth of rainbow trout fed SBM-based diet.
